# Dopaminergic modulation of pancreatic beta-cell insulin secretion and implications for antipsychotic-induced glucose dysregulation: a systematic review and meta-analysis

**DOI:** 10.1038/s41386-026-02388-0

**Published:** 2026-04-20

**Authors:** Wiaam Al-Hasani, Thomas Cheliotis-James, Anthony S. Wierzbicki, Zachary Freyberg, Oliver Howes, Toby Pillinger

**Affiliations:** 1 Department of Psychosis Studies, Institute of Psychiatry, Psychology, and Neuroscience, King’s College London, London, UK; 2 Guy’s and St Thomas NHS Foundation Trust, Conybeare House, London, UK; 3 Department of Psychiatry, University of Pittsburgh, Pittsburgh, PA USA; 4 Department of Cell Biology, University of Pittsburgh, Eye and Ear Institute, Pittsburgh, PA USA; 5 Imperial College London, London, Hammersmith Campus, London, UK; 6 South London and Maudsley NHS Foundation Trust, London, UK

**Keywords:** Medical research, Biochemistry, Diseases

## Abstract

Antipsychotic drugs exert their therapeutic effects through dopamine D_2_-like receptor blockade but are also associated with clinically significant dysglycaemia. Whether these metabolic effects reflect consistent actions on peripheral dopaminergic targets, including pancreatic β-cell D_2_-like receptors, remains uncertain. We conducted a systematic review and meta-analysis of preclinical in vitro and ex vivo studies examining the effects of dopamine, D_2_-like receptor agonists and antagonists on insulin secretion in isolated pancreatic islets or β-cell lines. PubMed, Embase, and PsycINFO were searched from inception to Sept 22, 2025. Two reviewers independently screened studies, extracted data, and performed random-effects meta-analyses with subgroup and meta-regression analyses assessing glucose concentration, compound type, and dose. 39 studies met inclusion criteria, with 37 included in the metanalysis. Dopamine and D_2_-like receptor agonists showed no significant effect under low-glucose conditions but robustly inhibited glucose-stimulated insulin secretion (GSIS) in rodent and rabbit models (g = –2.36; 95% CI: –2.77 to –1.96; *P* < 0.0001 & g = −1.98; 95% CI − 2.88 to −1.09; p < 0.0001 respectively), with greater GSIS suppression at higher glucose concentrations and dopamine doses. Though D_2_-like receptor antagonists alone had no significant effect (g = –0.25, 95% CI –0.68 to 0.18, *P* = 0.25), these drugs blocked GSIS inhibiton by co-administered dopamine (g = 1.59 [0.76 to 2.42]; p = 0.0002). These findings demonstrate that D_2_-like receptor activation inhibits pancreatic β-cell insulin secretion in a glucose- and dose-dependent manner, whereas receptor blockade reverses this effect, identifying a peripheral dopaminergic mechanism that may contribute to antipsychotic drug-associated dysglycaemia independent of weight gain. Together, these findings highlight the need for metabolic monitoring beyond weight alone in response to treatment with antipsychotic medications.

## Introduction

Antipsychotic drugs are cornerstone treatments for psychotic disorders, yet their metabolic consequences contribute substantially to the excess mortality seen in severe mental illness [1, 2]. Among these adverse effects, dysglycaemia is particularly important, characterised by insulin resistance and impaired glucose tolerance leading to type 2 diabetes (T2DM) [3, 4]. Real-world studies indicate a three-fold increase in T2DM risk among people prescribed antipsychotic drugs [5]. Although this dysglycaemia is often attributed to antipsychotic drug-induced weight gain, [6] growing evidence points to additional weight-independent effects on glucose metabolism [7]. Understanding the mechanisms driving these metabolic disturbances is therefore essential for improving the safety of existing treatments and guiding the development of new ones.

While antipsychotic drugs target a variety of receptors, nearly all exert their therapeutic effects through dopamine D_2_-like receptor blockade, raising the possibility that disrupted dopaminergic signalling contributes to their metabolic side-effects [8–10]. Importantly, we and others have shown that D_2_-like receptors are expressed not only in the brain but also in peripheral tissues, including the pancreas [10–12]. Converging evidence indicates that pancreatic β-cells synthesise dopamine which acts locally in an autocrine and paracrine manner to regulate glucose-stimulated insulin secretion (GSIS) [11, 13–15]. Supporting this, genetic polymorphisms of the dopamine D_2_ receptor are associated with insulin resistance and T2DM [16, 17]. Thus, D_2_-like receptor antagonism by antipsychotic drugs could disrupt dopaminergic modulation of insulin secretion, contributing to weight-independent metabolic side-effects. Clarifying whether insulin dysregulation is intrinsic to D_2_-like receptor blockade is therefore critical for understanding the metabolic risks of antipsychotic treatment. Consistent with this, preclinical studies indicate that D_2_-like receptor agonists and antagonists alter GSIS in both non-human and human pancreatic islets [10, 15, 18, 19]. However, the magnitude, direction, and consistency of these effects remain uncertain, and, to our knowledge, no meta-analysis has quantified the impact of dopaminergic agonism or antagonism on insulin secretion. To address this knowledge gap, we systematically evaluated D_2_-like receptor signalling and its effects on GSIS across preclinical models. Specifically, we aimed to: 1) quantify the effect of dopamine and selective D_2_-like agonists on insulin secretion in pancreatic islets and β-cell lines; 2) assess the consistency of these effects across species, tissue types, and glucose concentrations; 3) determine the impact of D_2_-like receptor antagonists on insulin secretion; and 4) identify sources of heterogeneity in dopaminergic modulation of β-cell insulin secretion.

## Methods

We performed a systematic review and meta-analysis in accordance with the PRISMA reporting guidelines. The review was registered on PROSPERO (CRD42024627599).

### Search strategy and selection criteria

We systematically searched PubMed, Embase, and PsycINFO from database inception to September 22, 2025 to identify preclinical experimental studies conducted in vitro or ex vivo that investigated effects of dopamine, or either selective D_2_-like receptor agonists or antagonists on insulin secretion. Eligible studies included those using isolated pancreatic islets or β-cell lines that reported quantitative measurements of insulin secretion under both basal low-glucose and stimulatory high-glucose conditions, in the presence or absence of D_2_-like receptor modulation. Studies were required to provide sufficient data to calculate effect sizes.

Studies conducted in vivo using whole animals were excluded because systemic confounding factors such as central, hormonal, autonomic, and pharmacokinetic influences could potentially obscure subtle β-cell-specific D_2_-like receptor effects to limit mechanistic interpretation. We also excluded studies that employed drugs targeting dopamine D_1_-like receptor subtypes (e.g., D₁/D₅ agonists or antagonists), as these are not the predominant targets of antipsychotic treatment. Studies using dopamine D_2_-like receptor antagonists with substantial affinity for other receptor systems known to modulate pancreatic β-cell insulin secretion (e.g., histaminergic H₁ and muscarinic M₃ receptors) were also excluded, based on reviewer consensus to minimise mechanistic heterogeneity and ensure that included drugs reflected primarily D_2_-like receptor-mediated mechanisms [9, 20, 21]. Additional exclusion criteria included the absence of a valid control group, lack of original data (e.g., review articles, commentaries), and publications not available in English without a full translation.

Search terms combined dopamine-related keywords with insulin-related terms using Boolean operators. The full search strategy is detailed in the Supplementary Materials. References identified from all databases were combined, and duplicates removed. Reference lists of review articles were also manually searched to identify additional relevant publications. Titles and abstracts were screened first, followed by full-text review of potentially eligible papers. Two reviewers (WA and TM) independently conducted the screening and full-text assessment, and discrepancies were resolved by consensus with a third reviewer (TP).

### Data extraction

WA and TM independently extracted data using a piloted standardised form. Extracted information included: study details (first author, year), experimental system (species, β-cell line, or isolated pancreatic islets), dopaminergic compounds (dopamine, D_2_-like receptor agonists/antagonists, dose), glucose concentrations, insulin assay type, and insulin secretion outcomes (mean absolute values or relative changes). Measures of variability and the number of experimental replicates were also extracted. Graphical data were digitised using WebPlotDigitizer [22]. Corresponding authors were contacted for studies deemed eligible but lacking sufficient quantitative data; a reminder was sent after two weeks if no response was received.

### Quality assessment

Methodological quality and risk of bias were assessed using the Quality Assessment Tool for In Vitro Studies (QUIN Tool), which evaluates key domains including selection bias, performance bias, detection bias, and reporting bias in preclinical experimental research [23].

### Statistical Analysis

We conducted random-effects meta-analyses using the metafor package in R [24] to pool effect sizes while accounting for expected between-study heterogeneity. Insulin secretion outcomes were expressed as standardised mean differences (Hedges’ g). Standardised mean differences were used to allow synthesis of data that used different measurement scales across studies [25]. Analyses were stratified by receptor activity to address several relevant biological questions: 1) dopamine or D_2_-like receptor agonists versus control (where control refers to islets or β-cell lines incubated under the same glucose conditions but in the absence of the respective dopaminergic agents), to test the physiological role of receptor activation; 2) D_2_-like receptor antagonists versus control, testing whether endogenous dopaminergic tone regulates insulin secretion; 3) D_2_-like receptor antagonists versus agonists (*i.e*., D_2_-like receptor antagonists in the presence of dopamine or D_2_-like receptor agonists), to test whether D_2_-like receptor blockade attenuates or reverses dopamine’s inhibitory effects on insulin secretion. A 2-tailed *P*  <  0.05 was deemed statistically significant. Because GSIS is glucose-dependent, studies were further stratified by glucose concentration: basal/fasting (2.5–5.6 mmol/L) versus stimulatory (8–55 mmol/L). Heterogeneity was assessed using Cochran’s Q and τ². Inconsistency was assessed using the I² statistic. Publication bias was evaluated with funnel plots and Egger’s regression test.

Sensitivity analyses assessed whether findings were consistent across experimental systems (e.g., islets, β-cell lines) and drug types (e.g., D_2_-like receptor agonists). First, results from isolated pancreatic islets and β-cell lines were compared using a Wald-type test to evaluate whether model type influenced insulin secretion outcomes. Second, experiments using selective D_2_-like receptor agonists were compared with those using dopamine or L-DOPA, which have broader receptor activity, to assess whether effects on insulin secretion were specific to D_2_-like receptor stimulation. Finally, meta-regression analyses were conducted to examine whether glucose concentration and dopamine dose were associated with changes in insulin secretion; a minimum of 10 independent experiments was required to perform meta-regression.

To minimise effects of potential biological heterogeneity between species, we pre-specified that rodent, human, rabbit and dog experiments would be analysed separately. However, only rodent and rabbit studies provided sufficient data for meaningful meta-analysis which was defined as a minimum of five methodologically comparable studies with sufficient reporting of the necessary quantitative information (i.e., group means with measures of variance and sample sizes) [26, 27].

## Results

Of 12,457 citations screened, 39 studies met inclusion criteria (see Table 1). Thirty rodent and five rabbit studies examined dopamine or selective D_2_-like receptor agonists, while 15 rodent studies also tested D_2_-like receptor antagonists. These studies met our predefined criteria for meta-analysis, as described above. One dog study and four human studies were identified in the review and were reported narratively. Common D_2_-like receptor agonists included dopamine, quinpirole, and bromocriptine; we also included the dopamine precursor L-DOPA in this category, though its modulatory actions on insulin secretion are primarily dependent on its conversion to dopamine [15]. Antagonists included haloperidol, sulpiride, and raclopride. Prisma Flow chart is presented in Fig. S10. Quality assessment scores are provided in Table S2. All included studies were classified as having a low to moderate risk of bias.

**Table 1:** Summary of studies included in the systematic review.

Author	Year	Animal / cell model	Agonist	Agonist Dose (s)	Glucose concentration at which Dopamine effect was tested (mmol/L))	Agonist compared to	D2/D3 Antagonist	Antagonist dose	Antagonist co-admistred with	Effect direction on insulin secretion
Rodent studies										
Al-Ghafari [51]	2022	CD1 mice beta cells	Not tested	NA	High = 25	NA	Haloperidol	10 µmol	Vehicle control	Haloperidol alone had no significant effect on (GSIS)
Arnerić [52]	1983	Sprague-Dawley rat islets	TL-99	1 µmol	High=16.6	Vehicle Control	Sulpiride	1 µmol	Epinephrine	TL-99 inhibits GSIS; Sulpride blocks the inhibitory effect of Epinephrine on insulin secretion
Asai [53]	2021	MIN6 cells	Dopamine	1,10,100, 100 µmol	High = 16	Vehicle Control	Not tested	NA	NA	Dopamine inhibits GSIS at all tested doses
Aslanoglou [19]	2022	BALB/c mouse islets, rat insulinoma-derived INS-1E cells	Bromocriptine, Quinpirole	1100 µmol	High = 20.0	Vehicle Control	Sulpiride	100 nmol	Bromocriptine	Bromocriptine & Quinpirole inhibit GSIS
Aslanoglou (2) [18]	2021	BALB/c mouse islets	Dopamine	1, 100 µmol	High = 20.0	Vehicle Control	Not tested	NA	NA	Dopamine inhibits GSIS; Sulpiride reduces dopaminergic inhibition of GSIS
Barrado [54]	2015	Wistar rat islets	Dopamine	1, 100 µmol	High = 10.0	Vehicle Control	Haloperidol	5 µmol	Dopamine, Vehicle control	Dopamine inhibits GSIS; Haloperidol reduces dopaminergic GSIS inhibition. Haloperidol alone has no effect compared to vehicle control
Csernus [55]	1998	Wistar rat islets	Dopamine	0.01 µmol	High = 55.0	Vehicle Control	Not tested	NA	NA	Dopamine inhibits GSIS
de Leeuw van Weenen [56]	2010	Rat insulinoma INS-1E cells	Bromocriptine	0.5, 0.05, 0.005, 0.0005 µmol	High = 20.0; Low = 2.5	Vehicle Control	Not tested	NA	NA	Bromocriptine inhibits GSIS
García-Tornadú [57]	2010	C57BL/6 J mouse islets	Dopamine	1,10 µmol	High=25	Vehicle Control	Sulpiride	10 µmol	Dopamine	Dopamine inhibits GSIS; Sulpride reduces dopaminergic GSIS inhibition
Farino [15]	2020	Rat insulinoma INS-1E cells	L-DOPA, Quinpirole	100 µmol for L-DOPA, 100 µmol for Quinpirole	High = 20.0	Vehicle Control	Raclopride	Log(-4)M	L-DOPA	L-DOPA, Quinpirole inhibit GSIS; Raclopride reduces agonist-induced GSIS inhibition
Ferrero [58]	2024	Rat insulinoma INS 832/13 cells	Dopamine, Quinpirole	10 µmol Dopamine; 0.1, 1 µmol Quinpirole	High = 11.0; Low = 2.5, 5.5	Vehicle Control	Haloperidol	10 µmol	Quinpirole	Dopamine and Quinpirole inhibit GSIS at 5.5 & 20 mmol/L glucose; Haloperidol reduces GSIS inhibition
Itoh [59]	1982	Wistar rat islets	Dopamine	2 µmol 20 µmol	High = 8.0	Vehicle Control	Haloperidol	5 µmol	Vehicle control	Dopamine inhibits GSIS; no significant effect for Haloperidol compared to vehicle control
Lebovitz [60]	1974	Egyptian sand rats	Dopamine	100 µmol	Low = 3.3	Vehicle Control	Not tested	NA	NA	No significant effect of dopamine on insulin secretion at low glucose
Lindstorm [61]	1982	ob/ob mouse islets	Dopamine, L-DOPA	400 µmol, 4000 µmol	High = 20.0	Vehicle Control	Not tested	NA	NA	Dopamine, L-DOPA inhibit GSIS
Lindstorm (2) [62]	1983	ob/ob mouse islets	L-DOPA	4000 µmol	High = 20.0	Vehicle Control	Not tested	NA	NA	L-DOPA inhibits GSIS
Liu [39]	2020	Wistar rat islets	Dopamine, Quinpirole	1, 10, 30 umol Dopamine; 10 µmol Quinpirole	High = 16.7, 8.3; Low = 2.5	Vehicle Control	Eticlopride	10 µmol	Vehicle control, Dopamine	Dopamine, Quinperole inhibit GSIS with no significant effects under low glucose condition; Eticlopride reduces dopamine’s GSIS inhibition, but has no significant effect alone
Maffei [28]	2015	Rat insulinoma INS-1E cells	Dopamine	100 µmol	High = 15.0	Vehicle Control	Not tested	NA	NA	Dopamine inhibits GSIS
Mahony [63]	1977	Golden hamster islets	Dopamine, L-DOPA	100 µmol	High = 16.5	Vehicle Control	Not tested	NA	NA	Dopamine, L-DOPA inhibit GSIS
Melkersson [64]	2001	Wistar rat islets	Not tested	NA	High = 16.7	NA	Haloperidol	1 µmol	Vehicle control	No signfincant effect of Haloperidol on insulin secretion compared to vehicle
Melkersson (2) [65]	2004	Wistar rat islets	Not tested	NA	High = 16.6	NA	Haloperidol	1 µmol	Vehicle control	No signfincant effect of Haloperidol on insulin secretion compared to vehicle
Nagata [66]	2019	Rat insulinoma INS-1E Cells	Bromocriptine Dopamine, HydroxyPIPAT	10,100 µmol Dopamine; µmol Bromocriptine; 10 µmol HydroxyPIPAT	High glucose concentration unspecified	Vehicle Control	Haloperidol, NGB2904	0.01, 0.03, 0.1 Haloperidol;0.001, 0.01 NGB2904	Vehicle Control	Bromocriptine, Dopamine inhibit GSIS, HydroxyPIPAT stimulatory under high glucose; Haloperidol blocks agonist actions; NGB2904 stimulates insulin secretion
Nogueira [67]	1994	Wistar rat islets	Dopamine	100 µmol	High = 16.7; Low = 5.6	Vehicle Control	Not tested	NA	NA	Dopamine inhibits GSIS and stimulates insulin secretion under low glucose conditions
Quickel [68]	1971	Golden hamster islets, Swiss-Webster mouse islets	Dopamine	10, 50, 100 µmol	High = 16.7	Vehicle Control	Not tested	NA	NA	Dopamine, L-DOPA inhibit GSIS
Rossini [69]	1973	Wistar rat islets	L-DOPA	250 µmol	High = 16.0	Vehicle Control	Not tested	NA	NA	L-DOPA inhibits GSIS
Rubí [11]	2005	Rat insulinoma INS-1E cells, Wistar rat islets, BALB/c mouse islets	Dopamine, Quinpirole	1, 10,100 Dopamine; 5, 10 µmol Quinperole	High = 15.0; Low = 2.5	Vehicle Control	Not tested	NA	NA	Dopamine, Quinpirole inhibit GSIS; Dopamine has no effect on insulin secretion under low glucose condition
Sridhar [70]	2024	Rat insulinoma BRIN-BD11 cells	L-DOPA	0.01, 1 µmol	High = 16.7	Vehicle Control	Not tested	NA	NA	L-DOPA inhibits GSIS
Szollosi [71]	2010	C57Bl/6 mouse islets	Dopamine	10 µmol	High = 15.0	Vehicle Control	Not tested	NA	NA	Dopamine inhibits GSIS
Takeuchi [72]	1990	Donryu rats islets	Dopamine	5.27, 15.27 µmol	High = 16.7	Vehicle Control	Domperidone	0.7 µmol	Dopamine	Dopamine inhibits GSIS at both doses. Domperidone reverses dopamine-induced GSIS inhibition
Uefune [73]	2022	C57BL/6 mouse islets	Dopamine	10 µmol	High = 16.7	Vehicle Control	Not tested	NA	NA	Dopamine inhibits GSIS
Ustione [74]	2012	C57Bl/6 mouse islets	L-DOPA, Quinpirole	0.1, 1, 3, 10, 100 µmol L-DOPA; 10 µmol f Quinpirole	High = 16.7; Low = 2.8	Vehicle Control	L741626, GR103691	5 µmol L741626; 1 µmol GR103691	L-DOPA	L-DOPA inhibits GSIS; L741626, GR103691 reduce L-DOPA GSIS inhibtion
Wei [75]	2018	C57BL/6 mouse islets	L-DOPA, Bromocriptine	5, 10, 25, 100 µmol for L-DOPA; 2,5 &10 µmol Bromocriptine	High glucose exact concentration unspecified	Vehicle Control	Sulpiride	10 umol	L-DOPA, Bromocriptine, Vehicle Control	L-DOPA, Bromocriptine inhibit GSIS; Sulpiride reduces GSIS inhibition by L-DOPA, but not Bromocriptine. Sulpride alone has no significant effect on GSIS compared to vehicle control
Yamaguchi [76]	1983	Wistar rats Islets	Dopamine	0.01, 0.1 µmol Dopamine	High = 16.7	Vehicle Control	Not tested	NA	NA	Dopamine inhibits GSIS
Zern [77]	1980	Golden hamster islets	L-DOPA	65 mg/Kg (ex-vivo study)	High = 16.6; Low = 3.3	Vehicle Control	Not tested	NA	NA	No effect on GSIS, inhibitory under low glucose condition
Rabbit studies										
Aleyassine [78]	1972	New Zealand rabbits	Dopamine	100 µmol	High = 16.5	Vehicle Control	Not tested	NA	NA	Dopamine inhibits GSIS
Feldman [79]	1974	Albino rabbits	L-DOPA	100,500 µmol	High = 16.5	Vehicle Control	Not tested	NA	NA	Dopamine inhibits GSIS
Feldman [79, 80]	1975	New Zealand & white rabbits	L-DOPA	10 µmol	High = 16.5	Vehicle Control	Not tested	NA	NA	L-DOPA inhibits GSIS
Quickel [68]	1971	New Zealand rabbits	Dopamine	10,50,100 µmol	High = 16.7	Vehicle Control	Not tested	NA	NA	Dopamine inhibits GSIS at all tested doses
Wilson [81]	1974	New Zealand rabbits	L-DOPA	50 mg/ kg (ex-vivo)	High = 16.5	Vehicle Control	Not tested	NA	NA	Dopamine inhibits GSIS
Human Studies										
Simpson [13]		Human islets	Dopamine, Quinpirole	1 umol	High = 16.7	Vehicle Control	Haloperidol, Sulpiride	10	Dopamine	Dopamine, Quinpirole inhibit GSIS. Haloperidol, Sulpiride reverses this effect
Maffiei [28]	2015	Human islets	Dopamine	50,100 umol	High =15	Vehicle Control	Not tested	NA	NA	Dopamine inhibits GSIS at both doses
Aslanoglou [18]	2021	Human islets	Bromocriptine	0.00001, 0.000001 µmol	High = 20	Vehicle Control	Not tested	NA	NA	Bromocriptine has no significant effect on GSIS at either dose
Aslanoglou [19]	2022	Human Islets	Dopamine	0.1, 1, 100 µmol	High = 20	Vehicle Control	Not tested	NA	NA	Dopamine inhibits GSIS
Dog Study										
Hermansen [30]	1978	Mongrel dogs	Not tested	NA	Low =1.4 High = 8.3	NA	Haloperidol	0.2, 4, 10 µmol	Vehicle control	Haloperidol inhibits insulin release at all concentrations

## Results of the meta-anlysis

### Effects of dopamine and D_2_-like receptor agonists on insulin secretion under low-glucose conditions

**Fig. 1:**
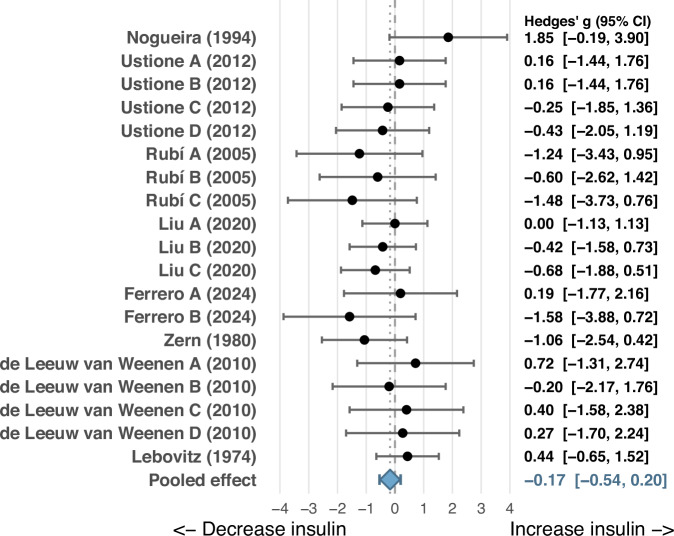
Forest plot showing effects of dopamine and D_2_-like receptor agonists on pancreatic β-cell insulin secretion under basal, low-glucose conditions (2.5-5.6 mmol/L). Labels A, B, C, D etc., denote independent experimental comparisons within the same study, reflecting different doses and/or different D2-like receptor agonists.

Nineteen experiments from eight rodent studies showed no significant effect of dopamine or D_2_-like receptor agonists on insulin secretion under basal, low-glucose (2.5–5.6 mmol/L) conditions (Hedges’*g* = –0.17; 95% Confidence Interval (CI): –0.54 to 0.19; *P* = 0.36; Fig. 1). Heterogeneity was negligible [*Q*(18) = 12.99, τ² = 0.00, *I*² = 0.0%].Visual inspection of the funnel plot showed no evidence of asymmetry and Egger’s test indicated no evidence of publication bias (Z = −0.25, p = 0.79). Trim-and-fill analysis did not identify any missing studies, and the pooled effect remained small and not significant (Hedges’ g = −0.17, 95% CI − 0.54 to 0.20)(Fig. S1).

Subgroup analyses and Wald-type tests comparing rodent β-cell lines (k = 6) and rodent pancreatic islet studies (k = 13) revealed no statistically significant difference in the effect of dopamine on insulin secretion under low-glucose conditions (*P* = 0.59). Likewise, subgroup analysis comparing studies that used dopamine or dopamine precursor L-DOPA (k = 14) with those using D_2_-like receptor agonists (k = 5) showed no significant difference between subgroups (*P* = 0.27). Meta-regression analyses did not provide evidence of an association between glucose concentration (K = 19) or dopamine dose (K = 10) and the suppressive effect of dopamine agonism on insulin secretion under these basal conditions (*P* = 0.98 & *P* = 0.07, respectively) (Figs. S2 & S3).

### Effects of dopamine and D_2_-like receptor agonists under high-glucose conditions

**Fig. 2:**
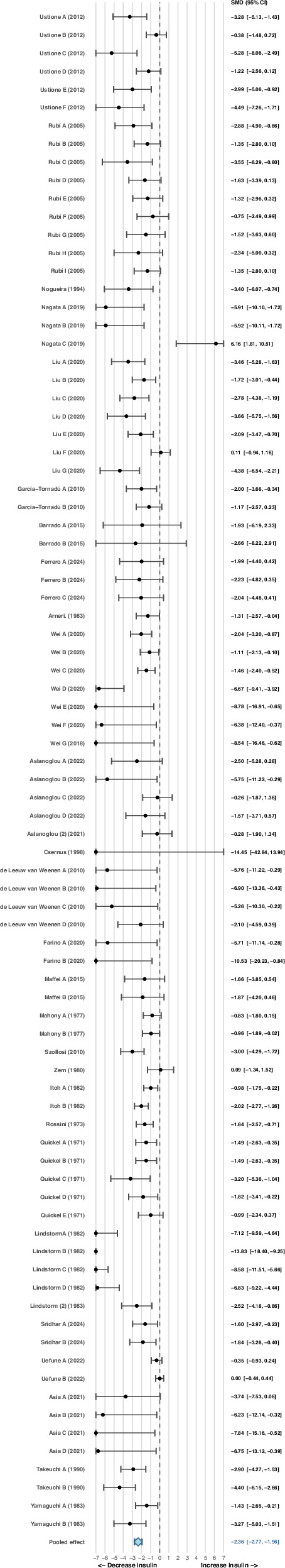
Forest plot showing the effects of dopamine and D_2_-like receptor agonists on insulin secretion at high-glucose conditions (8–55 mmol/L). Labels A, B, C, D etc., denote independent experimental comparisons within the same study, reflecting different doses and/or different D_2_-like receptor agonists.

Across 84 experiments from 29 rodent studies, dopamine and D_2_-like receptor agonists significantly reduced insulin secretion under high-glucose concentrations (*g* = –2.36; 95% CI: –2.77 to –1.96; *P* < 0.0001) (Fig. 2). Heterogeneity was high (Q (83) = 321.31, I² = 79.17%, τ² = 2.26. Although there was evidence of publication bias (Egger’s test: z = – 8.68, *P* < 0.0001), the inhibitory effect was robust to trim-and-fill adjustment (*g* = -−1.56, 95% CI –2.10 to –1.01, *P* < 0.0001) (Figure S4). Subgroup analyses using Wald-type tests showed no significant difference between β-cell lines (*k* = 34) and pancreatic islets (*k* = 50) (*P* = 0.19). Similarly, stratifying by drug type, comparing dopamine or L-DOPA (*k* = 61) with selective D₂-like receptor agonists (*k* = 23), revealed no significant subgroup difference (*P* = 0.56). Meta-regression demonstrated that both higher glucose concentrations (*k* = 73; *β* = –0.13 per 1 mmol/L increase; *P* = 0.038) and higher dopamine doses (*k* = 42; *β* = –0.0032 per 1 µmol/L increase; *P* < 0.0001) were associated with stronger inhibitory effects on insulin secretion during GSIS (Figs. S4 and S5).

Similarly, eight experiments from five rabbit studies showed that dopamine and L-DOPA exerted a significant inhibitory effect on GSIS (Hedges’ g = −1.98; 95% CI − 2.88 to −1.09; *p* < 0.0001). Between-study heterogeneity was also high (Q(7) = 24.13, I² = 72.2%, τ² = 1.18) (Figure S6). Egger’s regression test indicated potential publication bias (intercept = −4.29, *p* < 0.0001). Nevertheless, trim-and-fill analysis suggested that the adjusted pooled effect remained statistically significant (Hedges’ g = −1.75, 95% CI − 2.70 to −0.81, *p* = 0.0003) (Figure S7).

### Effects of dopamine antagonists on insulin secretion

**Fig. 3:**
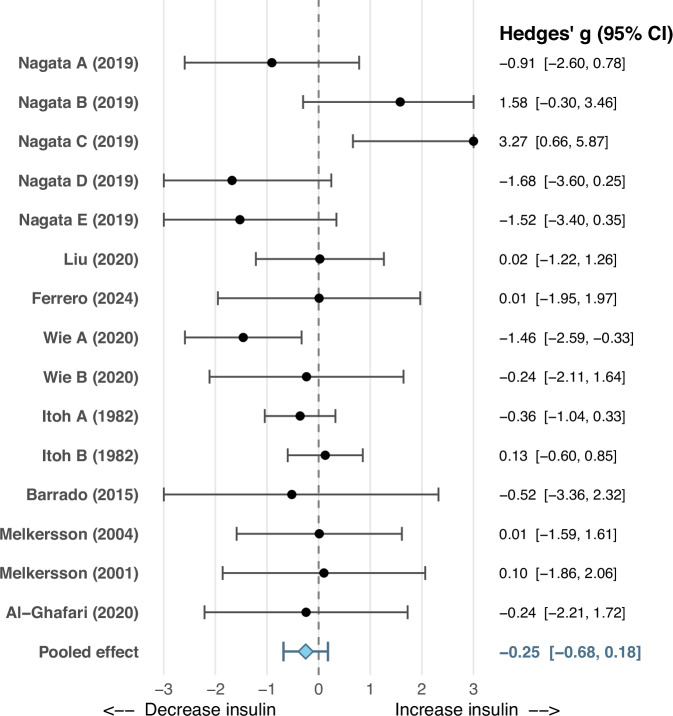
Forest plot showing the effect of D_2_-like receptor antagonists on insulin secretion compared to vehicle control. Labels A, B, C, D etc., denote independent experimental comparisons within the same study, reflecting different doses and/or different D_2_-like receptor agonists.

Fifteen experiments from nine rodent studies found no significant effect of D_2_-like receptor antagonists alone, compared to vehicle, on insulin secretion (Hedges’ g = –0.25, 95% CI –0.68 to 0.18, *P* = 0.25) (Fig. 3). Heterogeneity was low (Q(14) = 21.19, τ² = 0.14, I² = 22.78%). There was no evidence of publication bias (Egger’s test: z = 0.72, *P* = 0.46; Figure S8).

### Effects of D_2_-like receptor antagonists on dopamine-mediated inhibition of postprandial insulin secretion

**Fig. 4:**
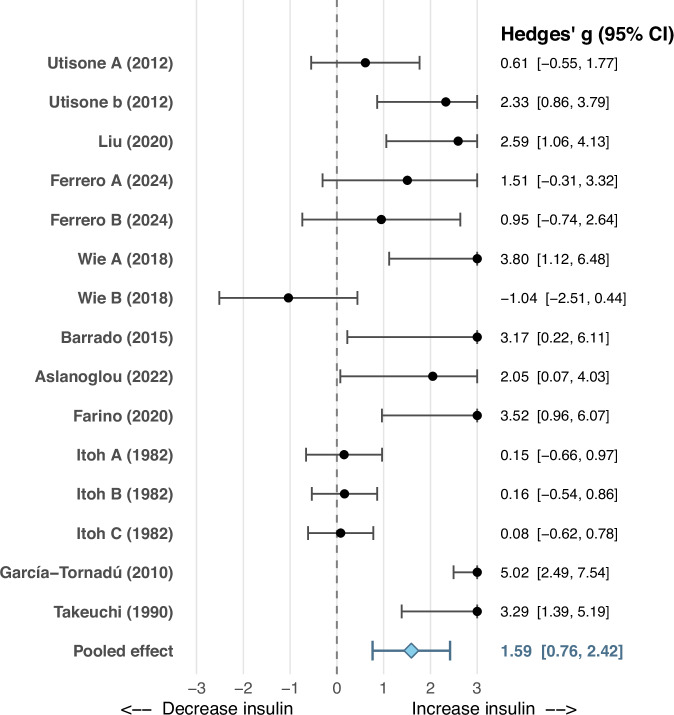
Forest plot for studies examining effects of D2-like receptor blockade by antagonists on insulin secretion in combination with agonist actions. Results are compared to dopamine agonism alone. Labels A, B, C, D etc., denote independent experimental comparisons within the same study, reflecting different doses and/or different D_2_-like receptor agonists.

Across 15 comparisons from ten rodent studies, co-administration of D_2_-like receptor antagonists administered alongside either dopamine or selective D_2_-like receptor agonists attenuated GSIS inhibition (increased insulin secretion) compared with agonist treatment alone (Hedges’ *g* = 1.59, 95% CI: 0.76 to 2.42, *P* = 0.0002) (Fig. 4). Heterogeneity was high (*Q*(14) = 56.23, *P* < 0.0001; τ² = 1.9, *I*² = 81%). Although Egger’s test suggested potential publication bias (Z = 5, *P* < 0.0001), the trim-and-fill analysis produced an adjusted effect (*g* = 0.85; 95% CI, –0.08 to 1.78; *P* = .079; *k* = 20) that was directionally consistent with the primary estimate (Figure S9). Subgroup analysis showed no significant difference between β-cell line and pancreatic islet studies in the effect of D_2_-like receptor antagonists on insulin secretion (*P* = 0.90). Meta-regression identified glucose concentration as a strong moderator of the effect of D_2_-like receptor antagonism on insulin secretion (K = 13, *P* < 0.0001), with each 1 mmol/L increase in glucose associated with a 0.21 unit increase in Hedges’ *g* (95% CI, 0.14–0.29) (Fig. S8). There were insufficient data to evaluate antagonist dose as a moderator of D_2_-like receptor antagonism on insulin secretion.

### Narrative summery of studies not included in the meta- analysis

Four studies examined dopaminergic modulation of insulin secretion in human islets under high-glucose conditions ranging from 15–20 mM. In human islets (Simpson 2012; Maffei 2015; Aslanoglou 2021, 2022) [13, 18, 19, 28, 29] dopamine and D_2_-like receptor agonists generally suppressed GSIS, whereas D_2_ antagonists increased insulin release. These findings support the presence of an inhibitory D_2_-like receptor-mediated negative feedback mechanism that regulates β-cell insulin secretion.

In contrast, one study conducted in dogs (Hermansen [30]) reported inhibition of insulin secretion by haloperidol across multiple concentrations under both low ( ≈ 1.4 mM) and high ( ≈ 8.3 mM) glucose conditions.

## Discussion

Our analyses show that dopamine and D_2_-like receptor agonists have no significant effect on pancreatic β-cell insulin secretion under fasting-range glucose concentrations, suggesting that dopaminergic tone does not meaningfully influence basal insulin release. In contrast, at glucose concentrations sufficient to depolarise β-cells to trigger GSIS, dopamine and D_2_-like receptor agonists produces a robust inhibitory effect, with the magnitude of inhibition increasing as both both glucose and dopamine concentrations rise. Moreover, D_2_-like receptor antagonists administered alone do not alter insulin seretion under either basal or glucose-stimulated conditions. This indicates little evidence of intrinsic or tonic D_2_-like receptor-mediated inhibition in the absence of dopamine [31]. However, when dopamine or selective agonists are present, antagonists substantially attenuate their inhibitory effects on insulin release. Together, these findings demonstrate robust glucose- and dose-dependent inhibitory effect of dopamine or agonists on pancreatic β-cell insulin secretion that is disrupted by D_2_-like receptor antagonism.

In the pancreas, dopamine is locally synthesised from L-DOPA taken up by β-cells, where it acts in an autocrine and paracrine manner via D_2_ and D_3_ receptors expressed on these cells [10, 32–36]. Our findings refine current understanding of β-cell GSIS regulation by showing that inhibitory dopaminergic signalling is glucose-dependent. The pronounced inhibition of insulin secretion under high glucose likely represents a physiological brake that prevents β-cell overstimulation and protects against metabolic stress [37]. This may also provide a therapeutic mechanism for bromocriptine, a D_2_-like receptor agonist and FDA-approved drug for treatment of T2DM [19, 38]. Conversely, the absence of a dopaminergic effect under basal, low-glucose conditions suggests minimal tonic dopamine receptor signalling in β-cells, with receptor activation occurring primarily in a glucose-dependent manner. Mechanistically, D_2_-like receptor activation couples to Gα_i/o_ proteins which inhibit adenylyl cyclase, lowering intracellular cAMP, and reducing calcium influx - thereby suppressing insulin granule exocytosis [39–42]. Activation of potassium channels further stabilises membrane potential, reinforcing this inhibitory cascade [39, 43]. Evidence from human studies aligns with these findings; however, meta-analysis was not feasible because only four human studies were identified, and one lacked sample size information.

The detection of D_2_-like receptor transcripts and associated Gα_i/o_ signalling machinery in both rodent and primate islets supports a shared molecular mechanism underlying dopaminergic modulation of β-cell excitability [11, 18, 44, 45]. Moreover, components of the dopamine biosynthetic and uptake machinery including tyrosine hydroxylase, aromatic amino acid decarboxylase and the dopamine transporter are expressed across species, enabling local dopaminergic feedback loops that fine-tune insulin output [11, 46, 47]. Collectively, these findings highlight the evolutionary conservation of dopaminergic regulation as a key feature of β-cell physiology.

Our findings suggest that acute antagonism of β-cell D_2_-like receptors attenuates agonist-induced inhibition of GSIS. Although the included studies do not address chronic exposure, we hypothesise that if such effects were sustained over time, they could contribute to hyperinsulinemia and the subsequent development of insulin resistance and impaired glucose tolerance. This mechanism provides a plausible pathway through which drugs with strong D_2_-like receptor antagonism, such as most antipsychotic drugs, may lead to dysglycaemia independently of effects on weight. Indeed, recent work has demonstrated that antipsychotic drugs cause significant disruptions in glycemic control independently of exposure time, dose, diagnosis, and weight gain propensity [7]. Conversely, D_2_-like receptor agonists used clinically, such as bromocriptine, reduce postprandial insulin and improve glycaemic control [48, 49] consistent with the inhibitory effects observed in our analysis. Together, these observations highlight pancreatic β-cell dopaminergic signalling as a potential target for understanding and mitigating metabolic side effects of antipsychotic drugs. In this context, xanomeline–trospium, an antipsychotic drug acting through muscarinic rather than dopaminergic mechanisms [50], represents an attractive therapeutic alternative with the potential to preserve metabolic function.

We acknowledge several study limitations. Variability in glucose concentrations, compound type, and dosing may have contributed to heterogeneity, and selective reporting cannot be fully excluded. Moreover, the relative scarcity of human islet data also limits the strength of direct translational inference. Certainty of evidence was not formally graded using the GRADE approach because this review exclusively included preclinical in vitro and ex vivo studies, for which GRADE is not routinely applicable.

Future studies using standardised experimental protocols and larger human datasets will be essential to validate these findings. There is also increasing recognition that, in addition to β-cells, other islet cell types including glucagon-secreting α-cells, also express D_2_-like receptors. Consequently, antipsychotic drugs also target α-cell dopamine receptors and disrupt dopaminergic regulation of islet glucagon secretion to further contribute to drug-induced glycemic control [18]. Thus, further studies are needed to more comprehensively describe the dopaminergic interplay between different islet cell types expressing D_2_-like receptors. Additionally, dopamine and D_2_-like receptor agonists like bromocriptine can not only signal through D_2_-like receptors but also via adrenergic receptors (e.g., α_2A_-adrenergic receptors) [18, 19], raising the possibility that drug signalling via islet adrenergic receptors can at least partially contribute to the changes in insulin secretion described here. However, relatively few studies within the scope of our review were designed to clearly disentangle these receptor families. Nevertheless, recent work where α_2A_-adrenergic receptors were genetically deleted from β-cells via a CRISPR/Cas9 technology revealed that while the overall efficacy of dopamine’s inhibition of GSIS was substantially reduced, its potency was substantially increased [18]. These data strongly suggest that α_2A_-adrenergic receptors and D_2_-like receptors play complementary albeit different roles in modulation of GSIS. The more abundantly expressed α_2A_-adrenergic receptors bind dopamine at lower affinities and thus likely primarily signal when local islet dopamine is in high abundance. On the other hand, the less abundantly expressed D_2_-like receptors bind dopamine at higher affinities compared to α_2A_-adrenergic receptors, providing a means to regulate GSIS under conditions when local dopamine concentrations are low. Future work is clearly needed to further disentangle the respective functions of α_2A_-adrenergic versus D_2_-like receptors in β-cells.

We note that many in vitro studies used micromolar dopaminergic concentrations that exceed physiological intra-islet levels [14]. While informative for receptor pharmacology, these doses may overestimate effects under physiological or clinical conditions. Finally, though the studies included here have exclusively focused on dopaminergic signalling in the pancreas as a putative driver for antipsychotic drug-induced dysglycaemia, we acknowledge that drug-induced disruptions of D_2_-like receptor signalling in metabolically-relevant brain regions (e.g., hypothalamus), also likely contribute to the development of dysglycaemia. Consistent with this, the targeting of both peripheral and central dopaminergic targets is required to improve dysglycaemia [38].

Despite these limitations, the study’s strengths lie in its receptor-specific focus and the integration of dose–response relationships under defined glucose conditions. This approach enabled systematic evaluation of D_2_-like receptor-mediated effects on β-cell function, linking receptor signalling to functional insulin output. Importantly, all included studies were assessed as having low to moderate risk of bias, supporting the internal validity of the findings. These findings provide a mechanistic framework for understanding antipsychotic-associated dysglycaemia. They underscore the need for metabolic monitoring that extends beyond weight, more informed antipsychotic selection to minimise metabolic risk, and investigation of peripheral dopaminergic modulation as a potential therapeutic approach to prevent or treat antipsychotic-induced glucose dysregulation.

## Conclusion

Activation of dopamine D_2_-like receptors suppresses GSIS in a glucose- and dose-dependent manner, while receptor blockade attenuates or reverses this effect. These findings identify dopaminergic signalling as a key regulator of β-cell function. Our results also suggest a potential mechanism linking peripheral dopamine D_2_-like receptor antagonism to disturbances in pancreatic β-cell insulin release that likely contribute to development of antipsychotic-induced dysglycaemia and T2DM.

## Supplementary information

SUPPLEMENTAL MATERIAL

S1

## Data Availability

All data used in this meta-analysis are derived from published studies. Extracted datasets and analysis code are available from the corresponding author upon reasonable request.
