# Supplementary material for: Dopaminergic modulation of pancreatic beta-cell insulin secretion and implications for antipsychotic-induced glucose dysregulation: a systematic review and meta-analysis
Source: Neuropsychopharmacology. 2026 Apr 20;51(7):1161–75. doi: 10.1038/s41386-026-02388-0 (PMC13212576; doi:10.1038/s41386-026-02388-0)
Supplement: Supplementary file 1 — SUPPLEMENTAL MATERIAL [file 41386_2026_2388_MOESM1_ESM.docx]

Supplementary Materials

**Dopaminergic modulation of pancreatic beta cell insulin secretion and implications for antipsychotic-induced glucose dysregulation: a systematic review and meta-analysis.**

Wiaam Al-Hasani FRCPath

Thomas Cheliotis-James

Anthony Wierzbicki DM DPhil FRCPath

Zachary Freyberg

Oliver Howes PhD

Toby Pillinger PhD

**^Search Strategy^**

We conducted a comprehensive literature search in Embase, MEDLINE, and PsycINFO via the Ovid platform, from database inception to 05 January 2026, with no language or publication date restrictions.

The search strategy combined terms related to dopamine signaling and insulin secretion/pancreatic islet function, The following search terms were applied to titles and abstracts (.ti,ab.):

Table (S1) Literature Search Strategy (Ovid Platform)

| **Search Set** | **Search Terms (Title/Abstract)** |
| --- | --- |
| **1. Dopamine-related terms** | dopamine OR dopaminergic OR “dopamine signaling” OR L-DOPA OR “dopamine transporter” OR DAT OR “dopa decarboxylase” OR “tyrosine hydroxylase” OR “monoamine oxidase” OR MAO OR “catechol-O-methyltransferase” OR COMT OR D1R OR D2R OR D3R OR D4R OR D5R OR DRD1 OR DRD2 OR DRD3* OR DRD4* OR DRD5* OR “dopamine agonist*” OR “dopamine antagonist*” OR antipsychotic* OR haloperidol OR raclopride OR bromocriptine OR sulpiride OR amisulpride |
| **2. Insulin and pancreatic islet-related terms** | insulin OR “insulin secretion” OR “beta cell*” OR islet* OR “islets of Langerhans” OR pancreas OR pancreatic |
| **3. Combined search** | 1 AND 2 |

**Update to quality assessment protocol.**

Although the protocol specified SYRCLE and NIH tools for quality assessment, it was subsequently noted that these instruments are primarily intended for in vivo animal and human studies. As the included studies were predominantly preclinical in vitro/ex vivo, we used the QUIN tool, which is specifically designed and validated for assessing risk of bias in preclinical in vitro research. This approach was therefore considered more appropriate for the study designs included.

**
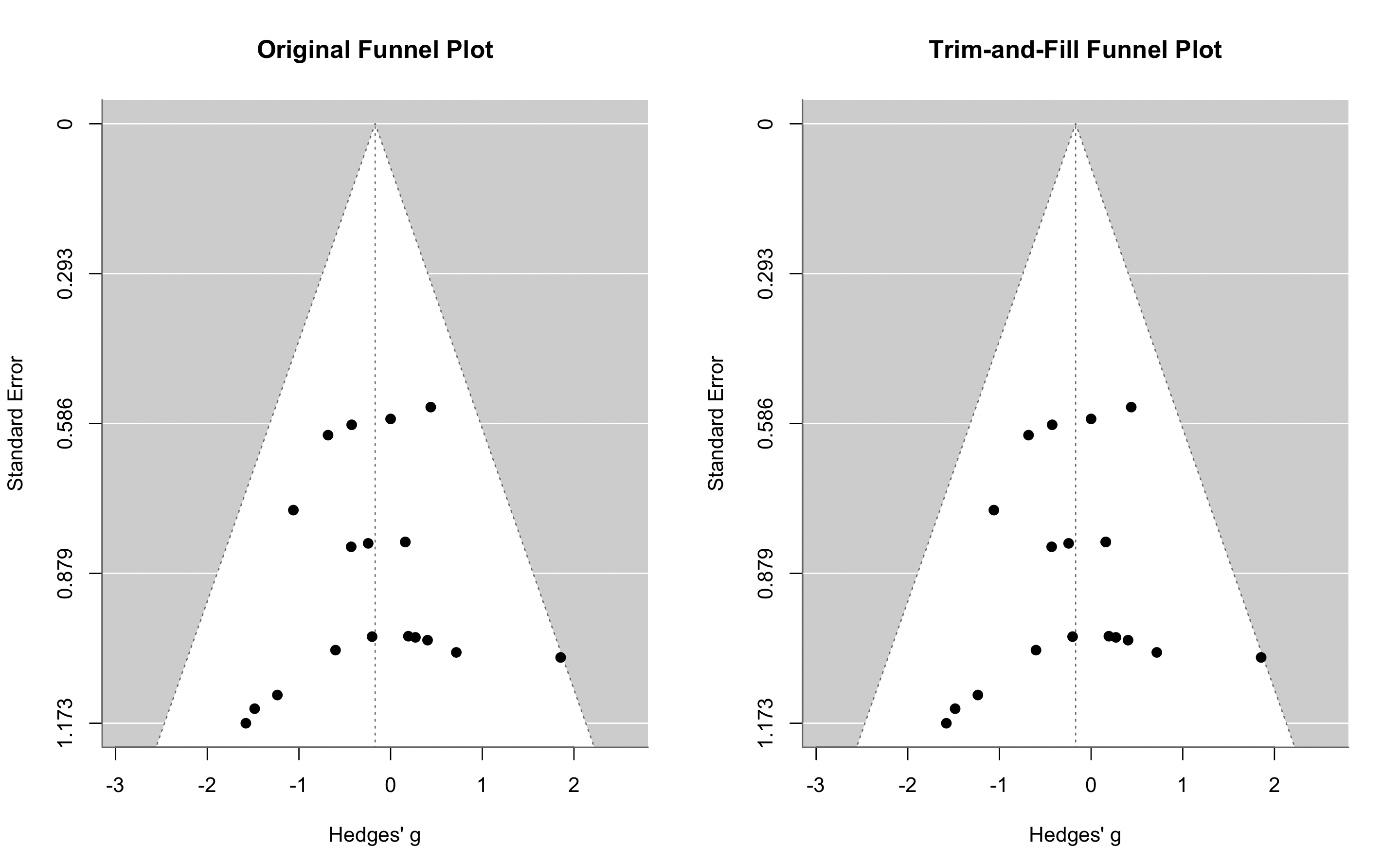
**

**Figure (S1):** original & trim and fill funnel plot for rodent studies looking at the effect of dopamine on insulin secretion at (fasting) low glucose level (2.5 to 5.6 mmol/L) compared to vehicle control.

**
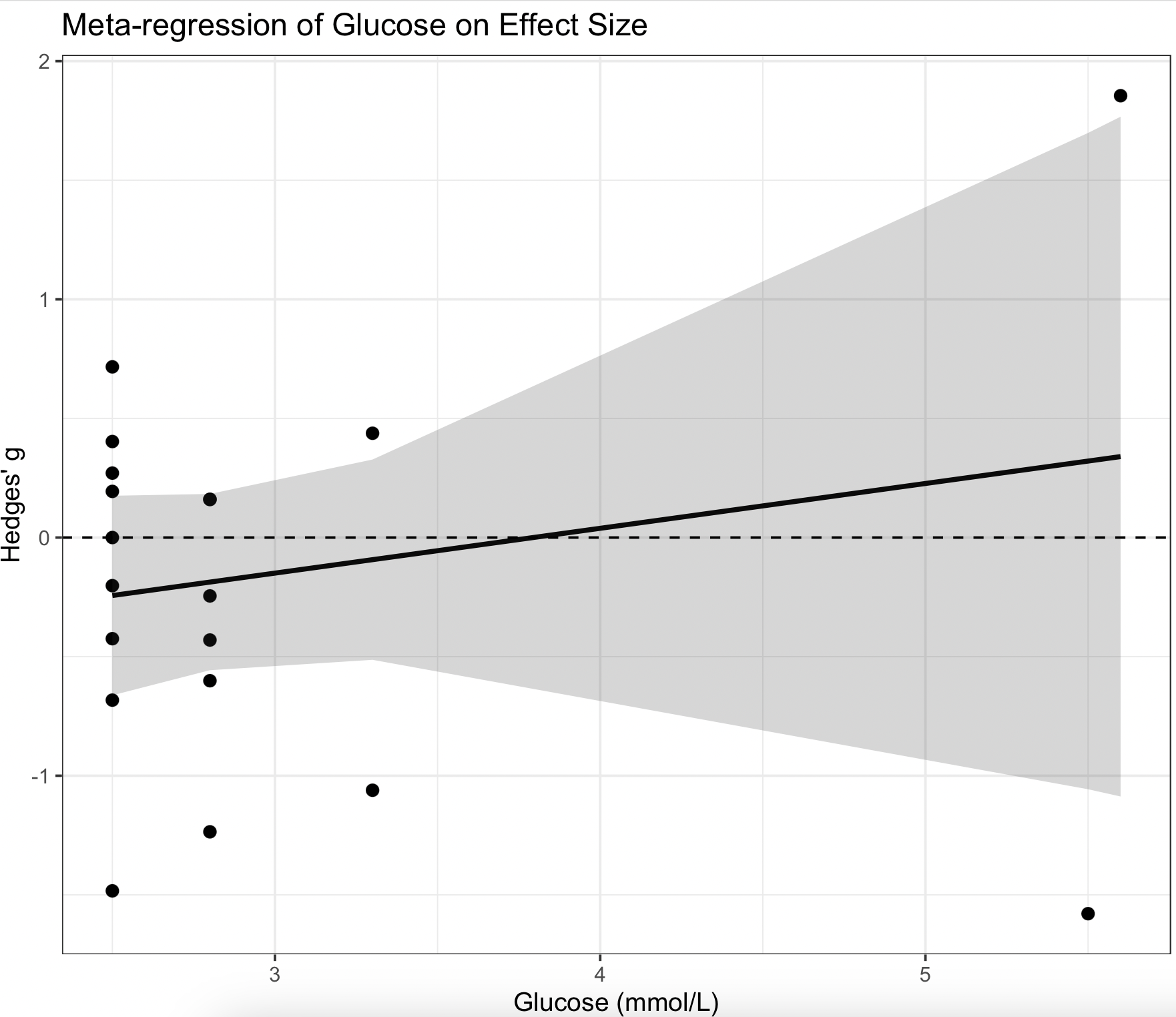
**

**Figure (S2):** Meta-regression of effect size for insulin secretion on glucose concentration (2.5 to 5.6 mmol/L) rodents solid line =regression line, shaded area =95% Confidence interval.

**
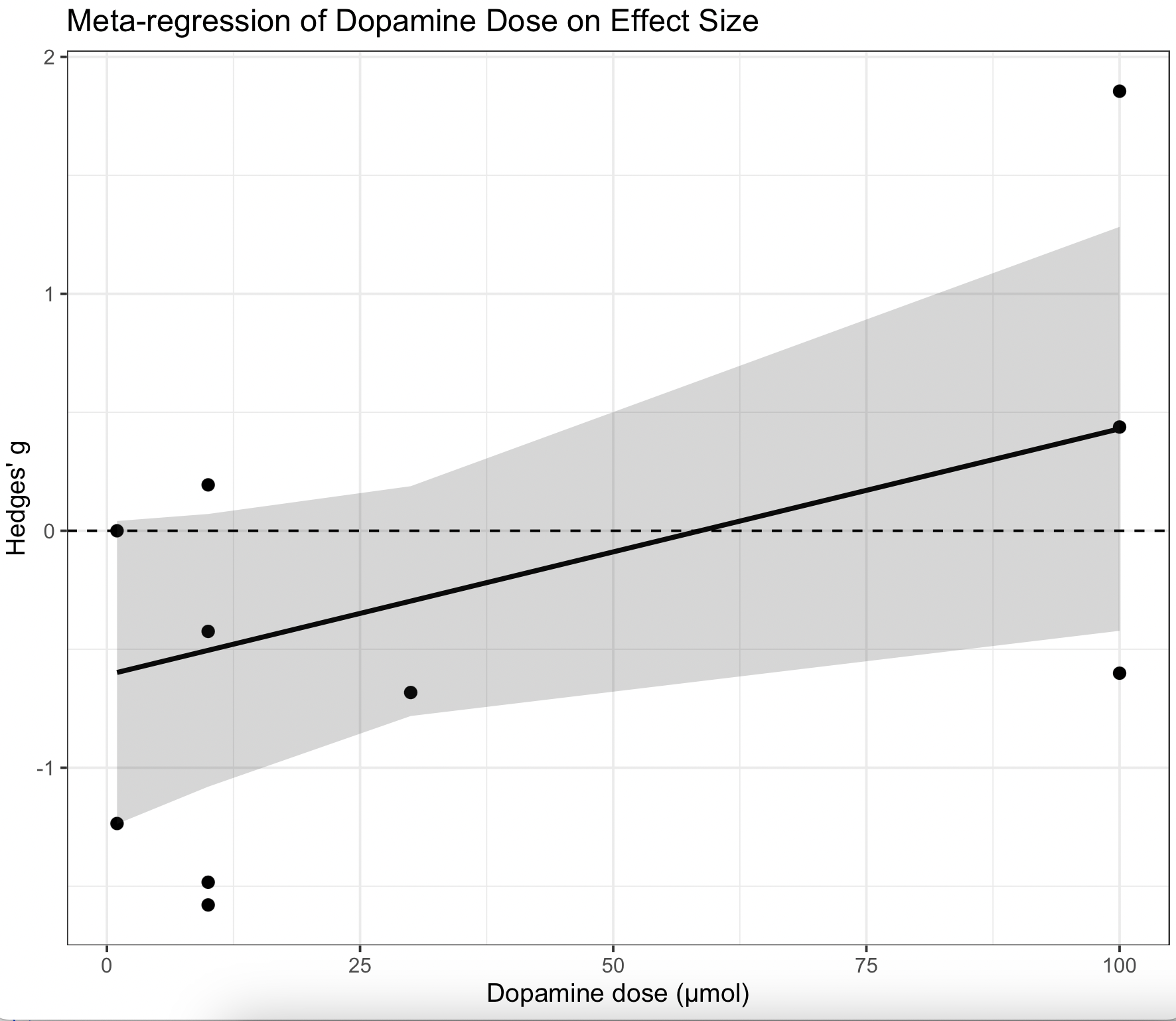
**

**Figure (S2):** Meta-regression of effect size for Dopamine dose on glucose concentration (2.5 to 5.6 mmol/L) in rodents solid line =regression line, shaded area =95% Confidence interval.

**
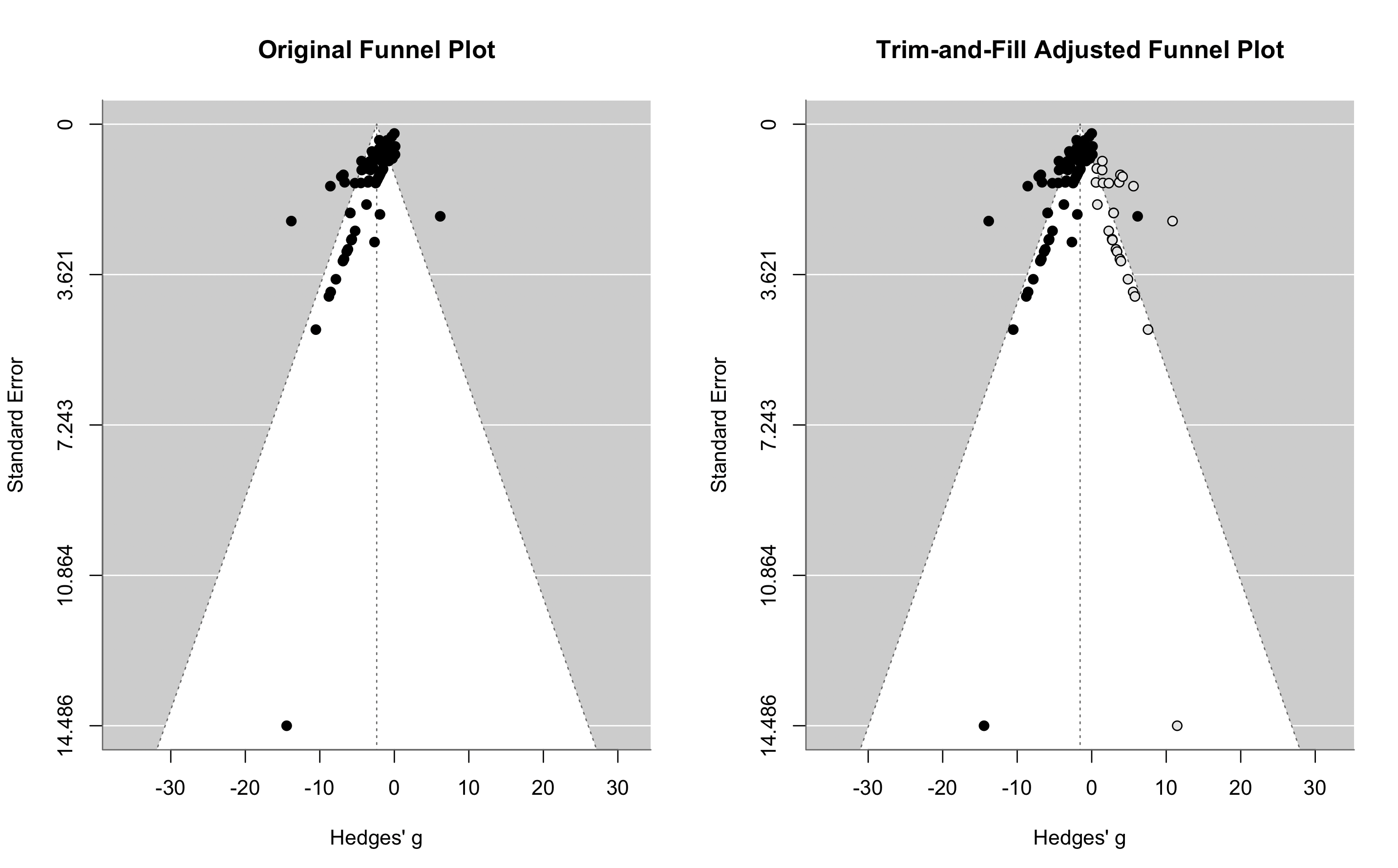
**

**Figure (S4):** Original funnel plot & Trim and Fill Funnel plot for studies looking at effect of dopamine on insulin secretion at high glucose level (8 to 55 mmol/L) compared to vehicle controls in rodents.

**
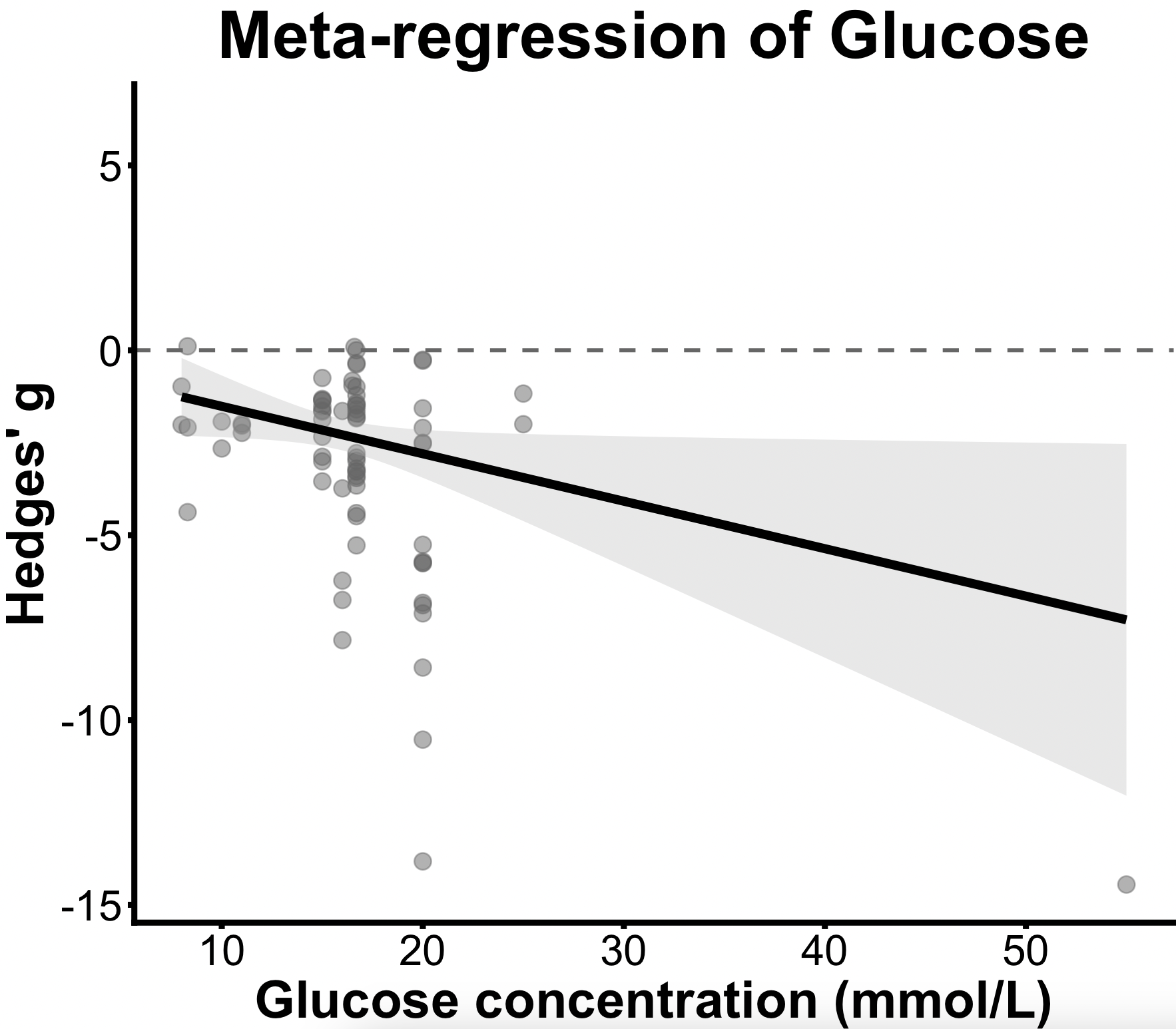
**

**Figure (S4):** meta-regression showing glucose concentration as a significant moderator of the effect of dopamine and D2/D3 agonists on GSIS in rodents . Exclusion of an extreme glucose concentration (55 mmol/L) did alter the observed association. The solid line represents the meta-regression estimate, and the shaded area indicates the 95% confidence interval.


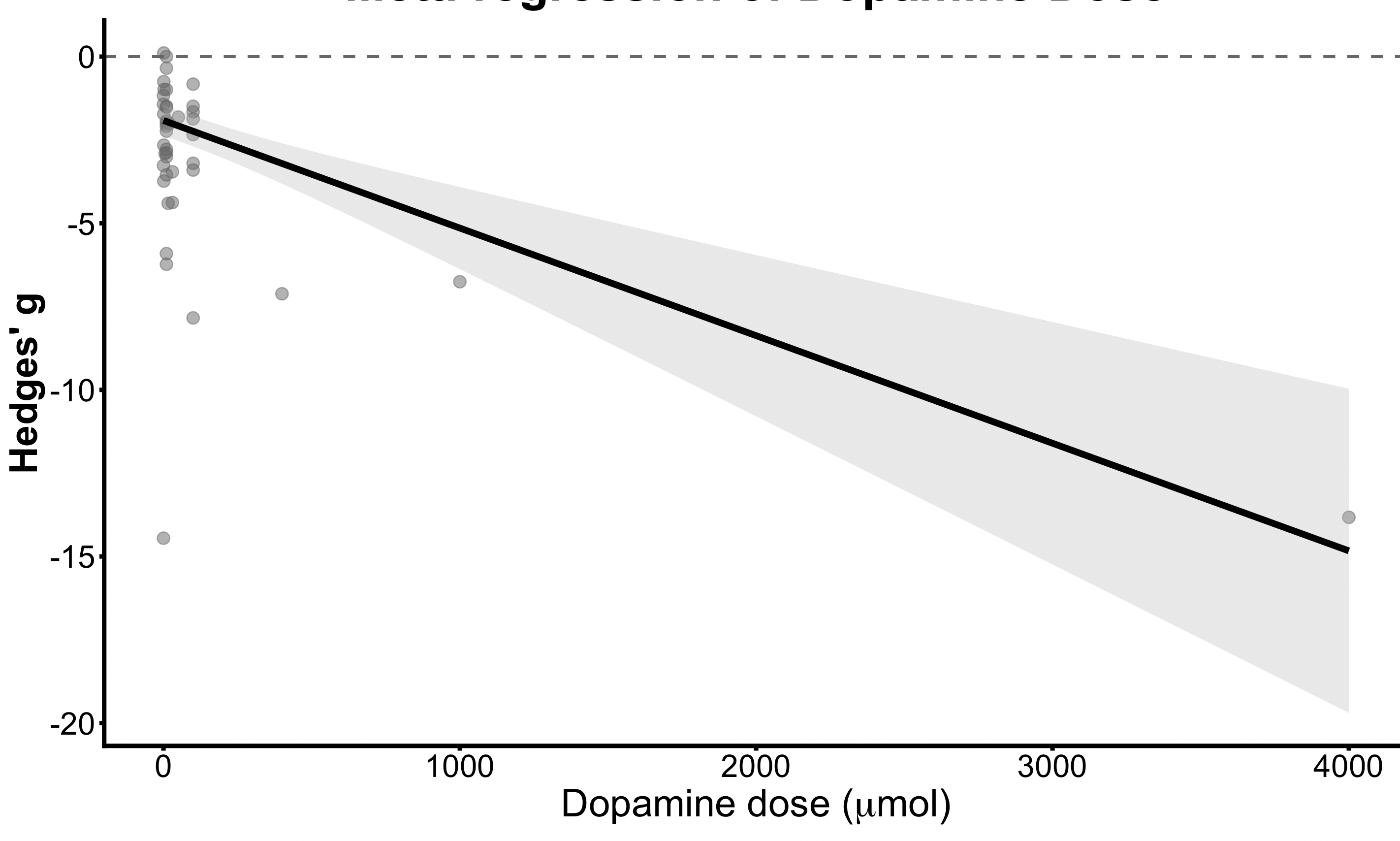


**Figure (S5):** meta-regression showing dopamine dose as a significant moderator of the inhibitory effect of dopamine on GSIS in rodents. Increasing dopamine dose was associated with a progressively stronger inhibitory effect. Exclusion of an extreme high dose (4000 µmol) did not alter the result. The solid line represents the meta-regression estimate, and the shaded area indicates the 95% confidence interval.

**
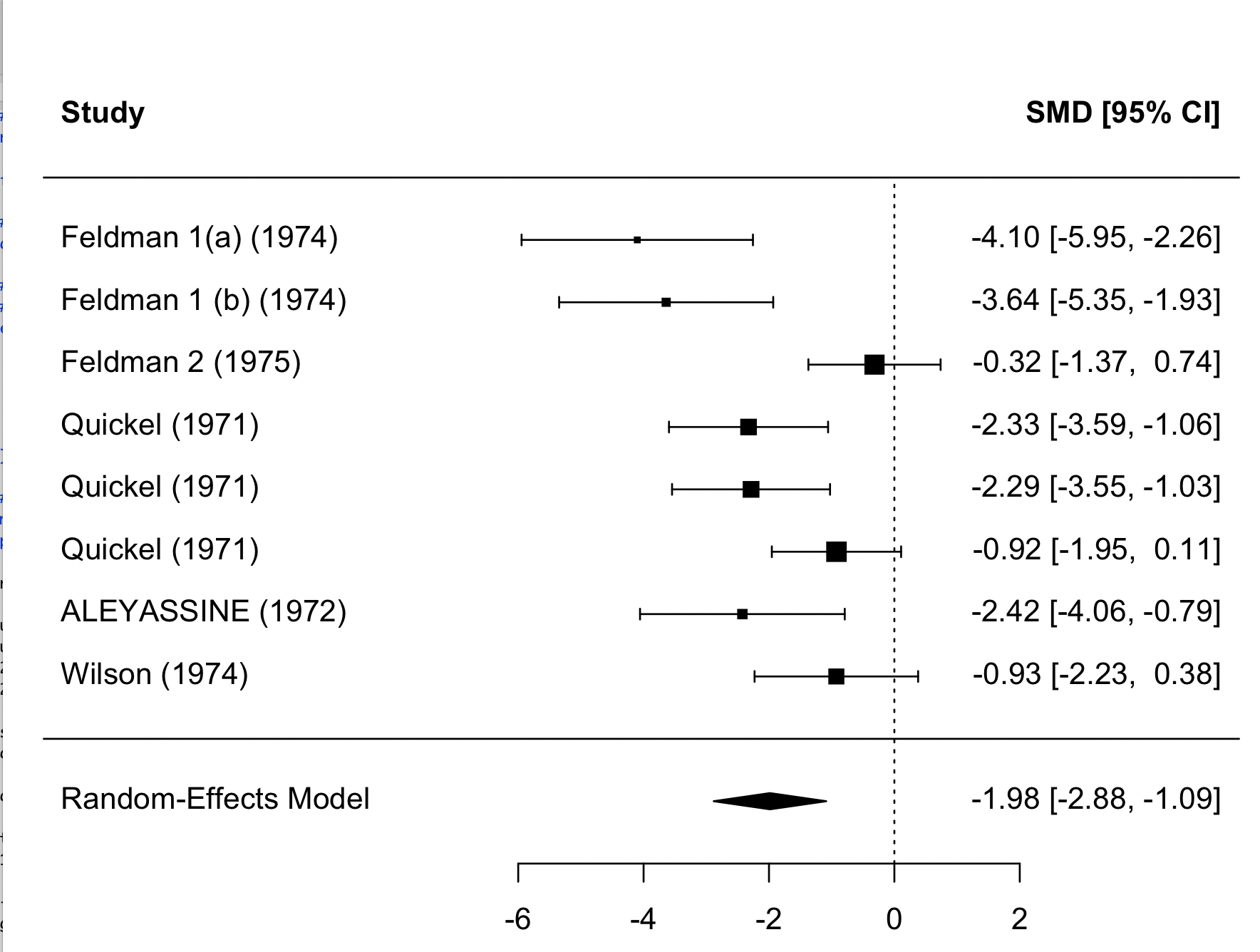
**

**Figure (S6):** Forest plot showing the effects of dopamine &L-Dopa on insulin secretion at high-glucose conditions (16.5-16.7 mmol/L) in rabbit models

**
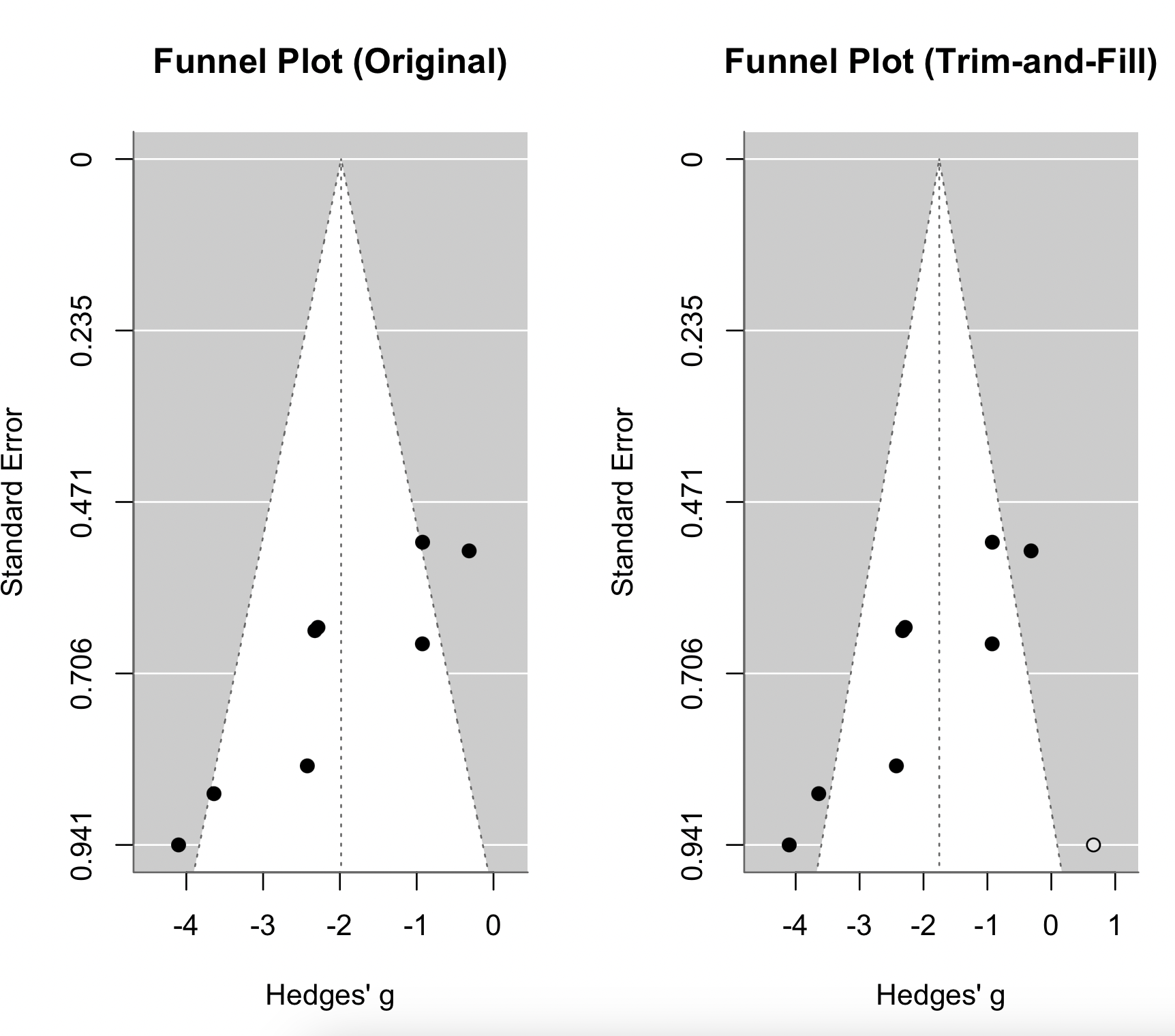
**

**Figure (S7):** Funnel plot for rabbit studies examining the effect of Dopamine on GSIS compared to vehicle.

**
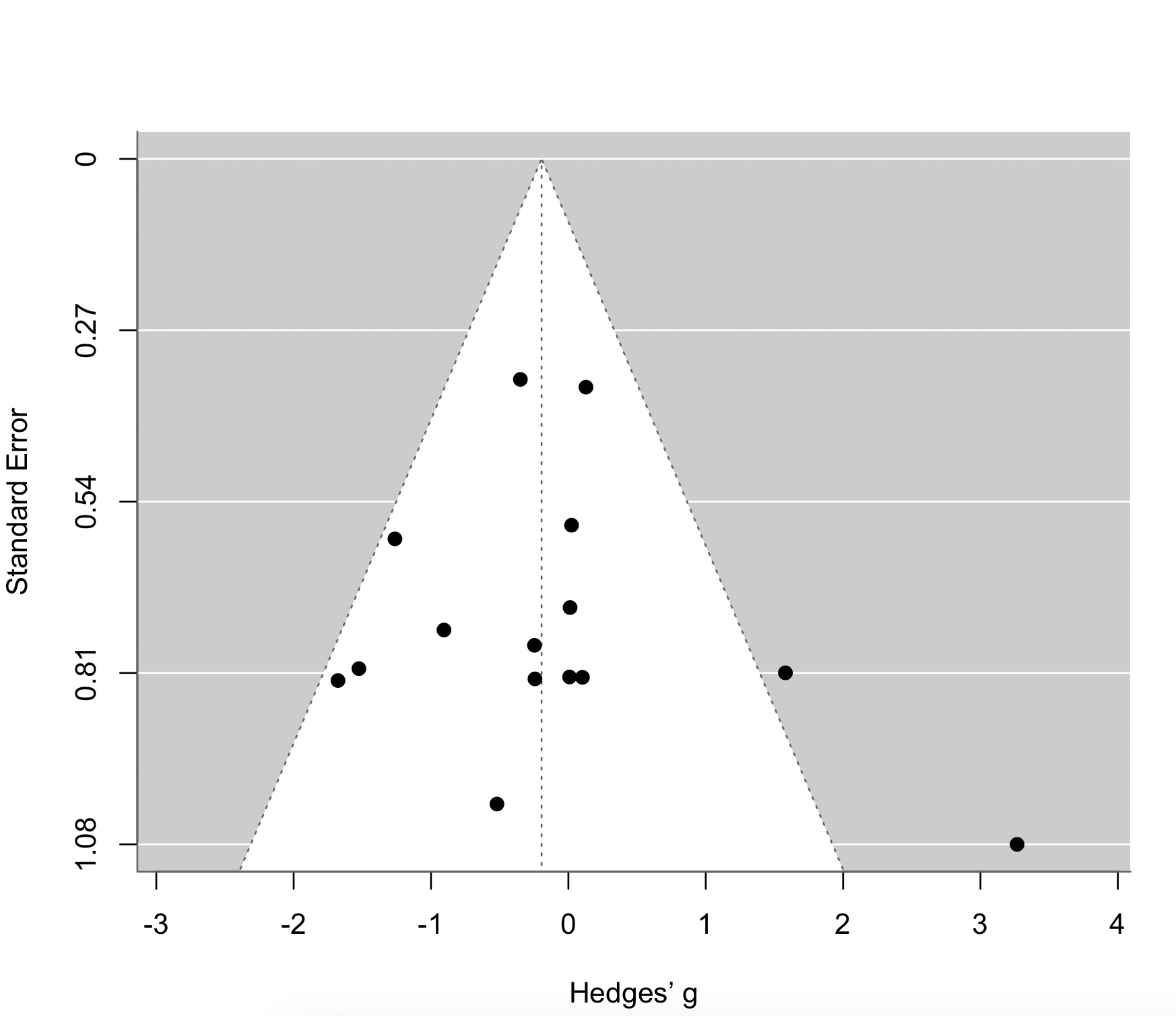
**

**Figure (S7):** Funnel plot for Rodent studies examining the effect of D2/D3 antagonists on GSIS compared to vehicle.

**
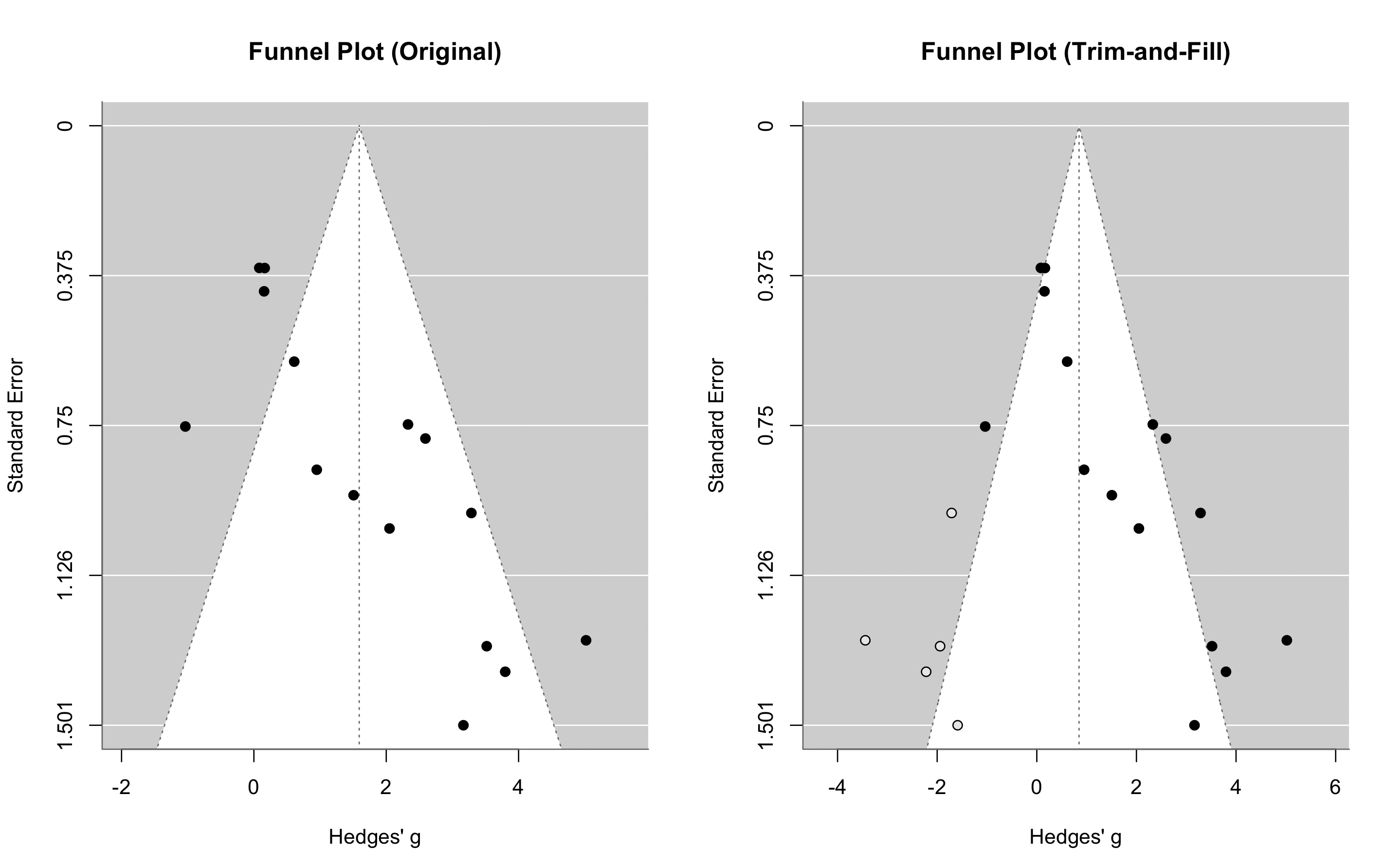
**

**Figure (S8):** Original funnel plot & Trim and Fill Funnel plot for studies looking at studies examining the effect of D2/D3 antagonists on GSIS compared to dopamine agonists

**
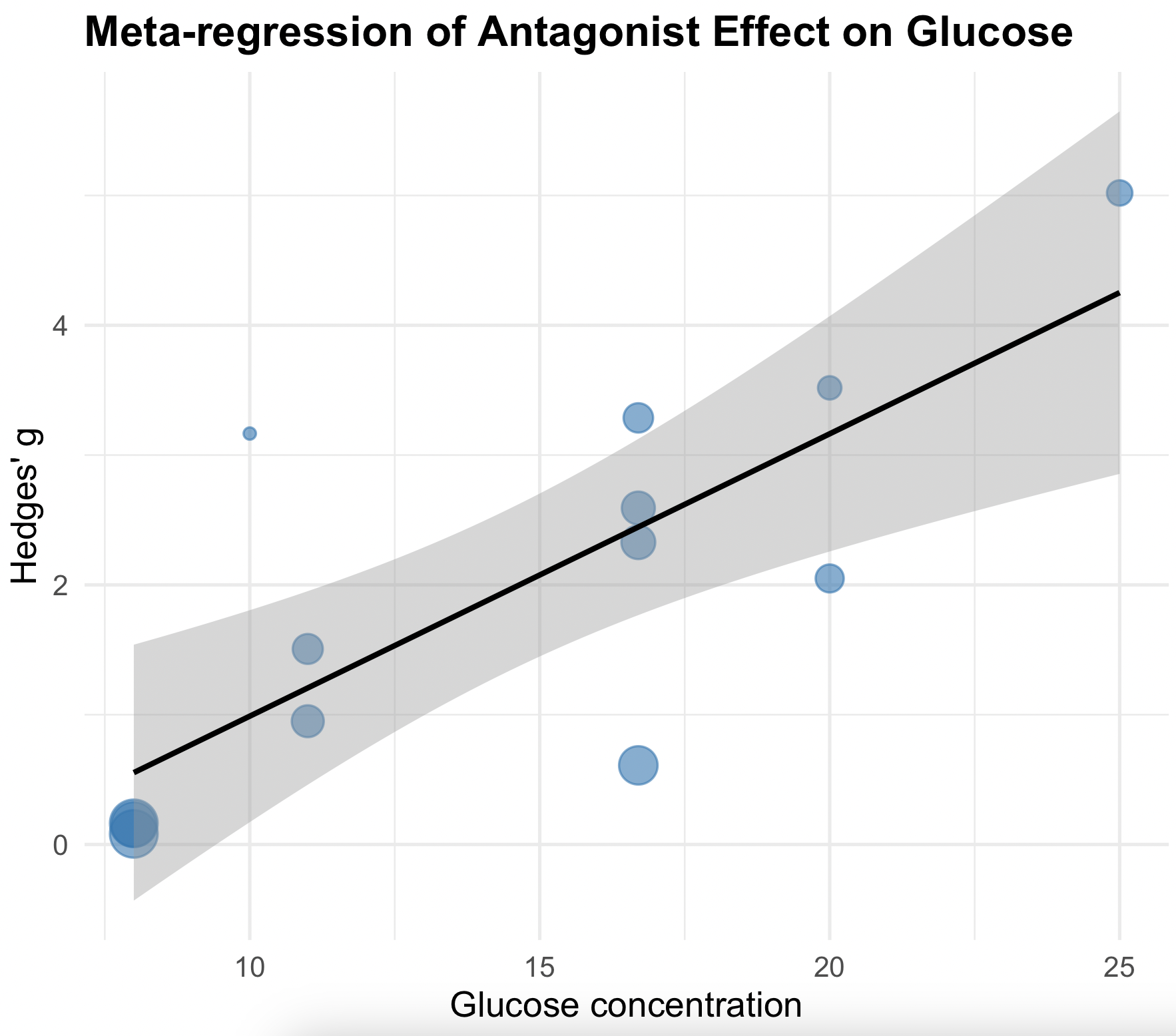
**

**Figure (S9):** meta regression showing glucose as a significant moderator for the effect of D2/D3 antagonists on dopamine-mediated inhibition of postprandial insulin secretion, solid line =regression line, shaded area 95% CI**.**

**Figure (S10):** PRISMA 2020 flow diagram

**Identification of studies via databases and registers**

Records removed *before screening*:

Records removed because no English translation available (n = 2679)

Duplicate records removed (n = 5003)

Records identified from ALL databases (n= 12457)

Embase (n = 6865)

Medline (n = 4614)

PsycINFO (n = 978)

**Identification**

Records screened

(n = 6775)

Records excluded after title abstract screening

(n = 6474)

Reports sought for retrieval

(n = 301)

Reports not retrieved

(n = 0 )

**Screening**

Reports excluded (n=262):

wrong outcome (n = 75)

wrong drug (n = 46)

wrong study design (n = 45)

in vivo studies (n = 32)

Histology studies (n = 21)

duplicate (n = 14)

Conference abstracts, no full text found (n = 13)

Central Dopamine (n = 6)

background article (n = 6)

Case report (n = 2)

No sample size (n = 1)

Other pharmacological drugs added to incubation medium (MAO inhibitor) (n = 1)

Reports assessed for eligibility

(n = 301)

Studies included in review

(n = 39)

**Included**
